# The Sin of So-Called Conservative Medical Treatment in Diseases Requiring Prompt Surgical Intervention

**Published:** 1903-05

**Authors:** Floyd Wilcox McRae

**Affiliations:** Atlanta, Ga.


ATLANTA

### Journal-Record of Medicine.

Successor to Atlanta Medical and Surgical Journal, Established 1855, and Southern Medical Record, Established 1870.

Vol. V. MAY, 1903. No. 2.

BERNARD WOLFF, M.D., EDITOR, Nos. 319–20 Prudential.

Published Monthly.

M. B. HUTCHINS, M.D., BUSINESS MANAGER, No. 64 Marietta St.

### *ORIGINAL COMMUNICATIONS.*

### THE SIN OF SO-CALLED CONSERVATIVE MEDICAL TREATMENT IN DISEASES REQUIRING PROMPT SURGICAL INTERVENTION.*

By FLOYD WILLCOX McRAE, M.D.,

Atlanta, Ga.

In choosing the above title, I realize fully that I lay myself and the surgical side of our profession open to criticism on the part of physicians who think we are too anxious to operate, and that we are constantly trying to find a good excuse to justify us in so doing. I appreciate further the fact that many unnecessary and useless operations are being done, have been done, and it is not my purpose to defend this kind of surgery. No physician could possibly have a greater contempt for a surgeon who would operate simply for a fee, or because the patient was willing to have some unneces-

*Read at the Columbus meeting of the Medical Association of Georgia.

sary thing done, than have I. I have been in a position to know of and see some surgery done that might better have been left undone. These, however, are but the exceptional cases, and I apprehend that there are comparatively few surgeons who in any way command the respect of even a small minority of their professional brethren in their immediate localities, who do much of this useless, and worse than useless, kind of work.

It is my purpose, however, to call the attention of the members of this Association to the sins of omission constantly committed by general practitioners in failing to call surgeons to help in the treatment of purely surgical conditions. It has been my misfortune, during the last few years, to see a great number of these neglected cases, that simple operations might have relieved had they been done early, which were practically hopeless when surgical aid was called in. I feel it my duty to call your attention to a few of these cases, as they were treated by physicians, and good physicians, who failed to realize the danger until it was too late, or to fully appreciate their responsibility as physicians. These mistakes have been the result of carelessness and inattention, rather than ignorance. Empyemas have been treated as typhoid pneumonias, strangulated femoral hernias as fecal impactions, and fulminant appendicitis as acute indigestion, because the doctors did not examine their patients carefully and arrive at a correct diagnosis by physical signs and exclusion. It has been my misfortune to operate on a number of cases of strangulated femoral hernia in women, the earliest time at which I was called in being four days after the hernia became strangulated. The other cases varied from five to eight days. In only one had a correct diagnosis been made, and that had been made by the patient herself, who was the widow of a physician. I operated on all these cases, but succeeded in saving only two out of six. Every one of these women ought to have been saved, and they could have been if early diagnoses had been made and immediate operations had been done. The innocent looking little tumor in the groin was considered of no importance and to have nothing to do with the case. Is it right to charge deaths like these to surgery? And still it is done by the community, and, I am sorry to say, not infrequently by the physicians themselves, who have not the moral courage to acknowledge their own mistake.

Every case of acute obstruction of the bowels should have the benefit at least of an early surgical consultation, whatever the cause of the obstruction ; whether it be a strangulated hernia, or some intra-abdominal condition. The symptoms are the same. There is pain, nausea, vomiting, collapse, weak, rapid pulse, cold, clammy skin, normal or subnormal temperature. Unless the obstruction be due to some acute inflammatory condition, the vomitus is first, the contents of the stomach, next, bilious matter, and then the intestinal contents. There may be one or two movements obtained from the lower bowel; after that, where the obstruction is complete, neither feces nor gas escapes. After fecal vomiting sets in and there is a rise in temperature due to beginning inflammatory changes in the intestine, operation offers comparatively little hope. It is a sin, not to say crime, to keep pouring down the throats of these unfortunate victims all sorts of nauseous and drastic purgatives in the effort to overcome a mechanical obstruction that can be relieved only by mechanical measures. Every attempt to purge such a patient increases the peristalsis, increases the pain, increases the exhaustion, increases the danger of the condition and lessens the chance of cure by the best planned and most skillfully executed surgical procedure.

Fulminant and perforative appendicitis are so frequently mistaken for, and treated as, acute indigestion, that I wish to call your attention to three of these cases that have fallen to my lot within the last fourteen months. One was a young man, sixteen years old, with a history of one or two mild attacks of appendicitis. He was taken suddenly, with violent pain, nausea, later vomiting; pain increased constantly, marked general depression, rapid pulse, leaky skin, but no temperature. The symptoms continued progressively increasing until noon of the fifth day, when there was a sudden violent pain, followed by extreme collapse, subnormal temperature, intense sweating, facies abdominalis marked, with the most extreme restlessness and an expression of anxiety which was distressing to see. I was called in late in the afternoon. Advised immediate operation. Patient was taken to St. Joseph's Infirmary and operated on twelve to eighteen hours after the onset of a general peritonitis. Pulse at the time of operation 140, temperature 97, respiration irregular, varying from 40 to 60. As soon as the peritoneal cavity was opened, a large quantity of foul pus spurted out; as the adherent coils of intestine were separated a general suppurative peritonitis was disclosed. The appendix had ruptured near the cecum, but the poison was shut off temporarily by adhesive inflammation, forming a large localized abscess which ruptured and flooded the general peritoneum. The patient died eighteen hours after operation.

The second case was a powerful negro mail-carrier, operated on at the Grady Hospital, with almost the identical history as the one above. I was assisted in this operation by Dr. Nicolson. An enormous gangrenous appendix had perforated, thin adhesions had formed about it, forming a localized abscess, which later ruptured, giving rise to a general septic peritonitis. He died about eighteen hours after the operation.

The third case was also operated on recently at the Grady Hospital. A prostitute had been treated for several months for attacks of pain and vomiting accompanied with fever, which would confine her to bed for from two to four days at each attack. She had been treated by a good gynecologist and by a general surgeon of considerable reputation, both of whom, according to her statement when she was brought to the hospital, had made a diagnosis of ovarian trouble and subjected her to prolonged local treatment. Operation disclosed a large, gangrenous appendix, free in the general peritoneal cavity, with just an adhesion of the tip to a small ovarian cyst, which had also become infected, forming an ovarian abscess. The most intense, septic peritonitis existed at the time of operation. Dr. Virgil O. Hardon was at the hospital and assisted me in this operation. We worked together, completing the operation in a very few minutes, during which time the patient was being infused with normal salt solution. In spite of the most vigorous stimulation subsequent to the operation, this poor girl died about forty-eight hours afterwards.

I had the opportunity of showing these last two cases to the graduating class of the Atlanta College of Physicians and Surgeons, and I hope the object-lessons which they presented will be the means of saving many lives. Nothing but a sense of individual responsibility could induce me to operate on cases such as the three just detailed. Only the exceptional one can be saved, but it is the surgeon's duty to give every one the possible chance. Had careful physical examinations been made, the local signs and constitutional manifestations received due consideration, surgical help called in, all of these individuals should have been saved. None of these cases was very violent to begin with, but all became hopeless through delay. Why have physicians and the laity such dread of the scalpel? What is more conservative than a well-planned, skillfully executed surgical operation? Is it right to charge surgery with deaths that are due solely to delay. In the name of all that is right and fair, No! Sooner far blame the doctor or the patient who had withheld the surgeon's life-saving hand! In this day of clean, scientific surgery the deaths chargeable to operations *per se* are too few to be recorded in comparison with those due to procrastination, until surgery is resorted to only as a forlorn hope. Medicine has failed ignominiously. Surgery—*dernier ressort* surgery—is saddled with untoward results that rightfully belong to medicine, sins of omission or commission.

I have operated on almost a moribund case of typhoid perforation, eighteen to twenty hours after the typical signs of perforation, only to find the small, clean-cut perforating ulcer, that might have been closed with two or three Lembert sutures, had eventuated in a general peritonitis.

I have seen many cases of osteomyelitis that had been treated for weeks medically, terminated by loss of life or limb.

How many good old men are suffering the tortures of the damned under medical or surgical palliative treatment, who might be cured by complete prostatectomies! I do not know of any operation that gives more complete relief, or any class of patients who are so appreciative as are these old men when they are made to urinate like boys again.

These are a very few of the surgical diseases that are mistreated medically. Their name is legion. Again I wish to disclaim any intention of exaggerating medical sins, or of minimizing those of surgery. We are all trying to get at the truth, to relieve pain, to prevent and cure disease, to lessen the sum total of human misery, to add to the years and the usefulness of our fellows.

With all the help we can get from physical signs, general symptoms, chemical and microscopical examinations of the urine, blood counts, X-ray examinations, etc., we must frequently be puzzled and make some mistakes. Other things being equal, mistakes will increase progressively as we fail to avail ourselves of these diagnostic aids. The symptomatic doctor, be he never so well read, who does not examine his patients, is a broken reed, upon which any poor sufferer must lean insecurely, finding no support when he most needs help.

What we all need is to take a broader, more comprehensive view of our professional obligations, to have less of rivalry, less of petty jealousies, to trust each other more completely, to consult more frequently and more freely, the physician with the surgeon, the surgeon with the physician, each with fairness and candor to the other, all with hearty zeal and unflagging interest in the God-like work of relieving human suffering, brightening the lives of those who trust us, putting ourselves in their places, doing the greatest good to the greatest number.

